# Duration of Breastfeeding, but Not Timing of Solid Food, Reduces the Risk of Overweight and Obesity in Children Aged 24 to 36 Months: Findings from an Australian Cohort Study

**DOI:** 10.3390/ijerph15040599

**Published:** 2018-03-26

**Authors:** Sarah Bell, Sarah Siau Yi Yew, Gemma Devenish, Diep Ha, Loc Do, Jane Scott

**Affiliations:** 1 School of Public Health, Curtin University, Perth, WA 6845, Australia; sarah.g.bell@postgrad.curtin.edu.au (S.B.); sarah.yew@postgrad.curtin.edu.au (S.S.Y.Y.); gemma.devenish@curtin.edu.au (G.D.); 2 Australian Research Centre for Population Oral Health, The University of Adelaide, Adelaide, SA 5000, Australia; diep.ha@adelaide.edu.au (D.H.); loc.do@adelaide.edu.au (L.D.)

**Keywords:** breastfeeding duration, solid food, complementary feeding, obesity

## Abstract

This study aimed to determine whether breastfeeding duration and the timing of solid food were independently associated with being overweight or obese in early childhood. Subjects were 953 children participating in the Study of Mothers and Infants Life Events Affecting Oral Health (SMILE) birth cohort study, based in Adelaide, Australia. Socio-demographic information and data on breastfeeding duration and age of introduction of solid food were collected at birth, 3, 4, 6, 12, and 24 months via mailed or online questionnaires completed by mothers. The weight and height of children were measured at a dental examination when children were aged between 24 and 36 months. Body mass index was calculated, and children were categorised into weight groups according to the World Health Organization growth standards. Multivariable logistic regression analysis was conducted, adjusting for maternal age at birth, education, socio-economic status, pre-pregnancy weight, smoking in pregnancy, method of delivery, and child’s birthweight. Risk of overweight/obesity was independently associated with maternal pre-pregnancy BMI, smoking in pregnancy, and birthweight. Children that were breastfed for 12 months or more had a significantly lower risk of being overweight/obese than those breastfed for less than 17 weeks (AOR 0.49; 95%CI 0.27, 0.90; *p* for trend =0.009). Age of introduction of solid food, however, was not associated with the risk of being overweight/obese at 24 to 36 months. This study provides further evidence of an inverse relationship between breastfeeding and risk of overweight/obesity, however, no association with the timing of solid food was detected.

## 1. Introduction

Obesity is a major public health problem that begins early in life, and it has been estimated that in 2016, 6% of children under the age of five years around the world were overweight or obese [1]. The prevalence is higher in high income countries, although the annual rate of increase is higher in low and middle-income countries [2]. The 2011–2012 Australian National Health Survey found that just under one in four (22.8%) of Australian children aged two to four years were overweight or obese [3]. These findings are concerning as obesity in childhood is associated with numerous immediate and long-term cardiovascular, metabolic, and psychosocial health outcomes [4], consequently placing the health system under increased economic stress. It has been estimated that after adjustment for significant maternal and sociodemographic characteristics, direct healthcare costs of obesity among Australian pre-schoolers are 62% higher than those of children with healthy weight [5].

Evidence from numerous systematic reviews suggests that breastfeeding is protective against obesity in childhood and later life [6,7,8,9,10]. As it is neither ethical nor feasible to conduct a randomised controlled trial (RCT) to investigate this effect, the evidence comes predominantly from prospective cohort studies. However, the results from these observational studies are not supported by the only cluster-RCT to explore the effects of breastfeeding among healthy term infants [11] and other more novel study designs (e.g., sibling-pair and cross-population) [12]. Therefore, the potential for residual and unmeasured confounding is large [13]. Studies have been criticised for not adequately controlling for measures of socio-economic status and other important factors, which have been shown to be associated with childhood obesity, such as infant birth weight [14], maternal pre-pregnancy body mass index (BMI) [14,15], smoking in pregnancy [14,16], and method of delivery [14,17]. 

Similarly, the influence that the age at which solid food is introduced has on future weight status has been investigated primarily in prospective cohort studies. However, in contrast to the observational evidence related to breastfeeding, no clear association between the age of introduction of solid food and childhood obesity has been found [14,18,19,20,21]. Several studies have reported an interaction between the age of introduction of solid food and duration of breastfeeding, with the early introduction of solid food (i.e., <4 months) being associated with increased weight gain only for infants breastfed for less than four to five months [22,23,24], but not in infants that were breastfed for longer. 

While the World Health Organization recommends that infants be exclusively breastfed to six months of age [25], there has been debate over the age at which solid food should be introduced to infants [26]. Nevertheless, there is general agreement amongst health authorities that exclusive breastfeeding to six months is desirable and that solid food should not be introduced before four months of age [27,28].

Currently, the Australian Infant Feeding Guidelines recommend exclusive breastfeeding for the first six months of life with appropriate solid food introduced around six months of age. Breastfeeding should be continued, while solid food is introduced until 12 months of age and beyond, for as long as the mother and child desire [29]. Despite these recommendations, the most recent Australian National Infant Feeding Survey found that only 15% of Australian infants are exclusively breastfed beyond five months and 35% consume solid food by four months of age [30].

To date, there have been relatively few Australian studies [31,32,33,34], which have investigated the association of breastfeeding and weight status in children and few have investigated concurrently the association of both age of introduction of solid food and the duration of breastfeeding [35]. The aim of this study, therefore, is to determine if breastfeeding duration and age of introduction of solid food are independently associated with being overweight or obese among a cohort of Australian children aged 24 to 36 months.

## 2. Materials and Methods 

This study analyses data collected from the Study of Mothers and Infants Life Events Affecting Oral Health (SMILE), which is a South Australian population-based birth cohort study of socio-economically diverse children [36]. The primary aim of SMILE is to identify and evaluate the relative importance and the timing of critical factors that shape the oral health of young children [36]. Mother-infant dyads were recruited from three metropolitan public hospitals in Adelaide, South Australia between July 2013 and August 2014. Recruitment typically took place within the first 48 hours after birth by trained health professionals who provided mothers with a written and verbal description of the study. The mothers were recruited consecutively and all newborns, regardless of birth weight and gestational age, were eligible to participate. Women whose English was insufficient to comprehend the written and verbal instructions and those that were living outside of the greater Adelaide area, or intending to relocate within the next 12 months, were excluded. 

Ethical approval for SMILE was obtained from the Southern Adelaide Clinical Human Research Ethics Committee (HREC# 50.13, approval date 28 February 2013) and the South Australian Women and Children Health Network (HREC# 13/WCHN/69, approval date 7 August 2013). Women were informed that their participation was voluntary and informed consent was obtained from all mothers who participated in the study. 

Socio-demographic information was collected at recruitment via self-completed questionnaires, and breastfeeding duration and the age at which solid food was introduced were derived from information collected via mailed or online questionnaires at ages 3, 6, 12, and 24 months. The weight and height of the children were measured at a dental examination which occurred between 24 and 36 months of age, using standardised methodology and equipment [36,37]. Questions used in this study to explore infant feeding practices were adapted from those used in the first and second Perth Infant Feeding studies [38,39] and which have been modified, translated and used widely in international studies [40,41,42,43].

The outcome of interest was the child’s weight status. Their BMI was calculated (kg/m^2^) and their weight status was then categorised, using the World Health Organization age and gender specific cut-offs, as healthy (BMI z-score ≥ −2 and ≤+2 standard deviations (SD)), overweight (BMI z-score > +2 and ≤+3 SD) or obese (BMI z-score > +3 SD) [44]. Due to the relatively small number of obese children, overweight and obese children were combined into a single category overweight/obese (BMI z-score > +2SD) for analytical purposes.

The primary explanatory variables of interest were breastfeeding duration and age of introduction of solid food. Breastfeeding duration was defined as the age at which an infant had ceased to receive any breast milk. Age cut points for categorising these two variables were based on ages identified in national [29] and international infant feeding guidelines [28] and used in other studies. As the timing of key feeding events was collected prospectively and recorded in weeks, 17, 26, and 52 weeks were considered to be the equivalent of 4, 6, and 12 months, respectively. Breastfeeding duration was categorised into less than 17 weeks, 17 to 25 weeks, 26 to 51 weeks, and 52 or more weeks, and the age of introduction of solid food was categorised into less than 17 weeks, 17 to 25 weeks and 26 or more weeks. 

Other known or putative risks factors for childhood obesity which were investigated and adjusted for included maternal age at child’s birth (years), level of education (high school or vocational, some university or above), country of birth (Australia/New Zealand, United Kingdom/Ireland, China, India, rest of Asia, other), number of children (1, 2, ≥3), pre-pregnancy BMI (<25 kg/m^2^, 25–29.99 kg/m^2^, ≥30 kg/m^2^), maternal smoking in pregnancy (yes, no), delivery method (vaginal, caesarean section), and infant sex and birth weight (per 100 grams). The socio-economic status of the family was determined using the postcode linked Index of Relative Socio-economic Advantage and Disadvantage [45], which was categorised into quintiles, where quintile 1 equals most disadvantaged and quintile 5 equals most advantaged.

All of the statistical analyses were conducted using SPSS version 24 (SPSS Inc., Chicago, IL, USA). The characteristics of participants and nonparticipants were compared using the Chi square statistic. Unadjusted and multivariable logistic regression was used to assess whether age of introduction of solid food and duration of breastfeeding were associated with overweight/obesity in children aged 24 to 36 months. The linearity of maternal age at birth and birth weight were confirmed in a preliminary analysis and these variables were examined as continuous variables, all other explanatory variables were examined as categorical variables. Secondary explanatory factors with an unadjusted *p* value of <0.20 were adjusted for in the multivariable model [46]. In total 878 children (92%) had complete data for all of the variables that were examined in the final multivariable logistic regression model. There was no pre-pregnancy BMI data available for 56 women (5.9%), and all other variables had less than 0.5% of data missing. Due to the small amount of missing data, no attempt was made to impute missing variables and cases with missing data for one or more of the variables entered into the multivariable logistic regression model were dropped from this analysis. To test for a possible interaction between the duration of breastfeeding and the age of introduction of solid food, the multivariable logistic regression analysis was repeated after stratifying the sample according to the breastfeeding status of children at 17 weeks. The level of statistical significance was set at *p* < 0.05.

## 3. Results

In total, 2181 children were recruited into SMILE, of which there were completed baseline questionnaires for 2112 children. The population used in the final analysis for this study consists of 953 children for which there were complete infant feeding data and a BMI z-score > −2 SD (Supplementary Figure S1). 

When compared with non-participants with baseline questionnaires (*n* = 1159), mothers of participant children were older, better educated, and more socially advantaged (Supplementary Table S1). The mean age of participant children at the time of the dental examination was 29.8 (±3.5 SD) months and the majority were normal weight (88.5%), had been breastfed for at least six months (26 weeks) (62.8%) and introduced to solid food before 6 months (90.8%) (Table 1).

In the unadjusted logistic regression analysis, infant birth weight was directly associated with risk of overweight/obesity and children were at greater risk of being overweight/obese if their mothers were in the most socially disadvantaged group (deciles 1–2 vs. deciles 9–10), had smoked in pregnancy (yes vs no), and were overweight or obese when they conceived (pre-pregnancy BMI ≥ 25 kg/m^2^ vs. <25 kg/m^2^). Children who had been breastfed for a year or more had a lower risk of being overweight/obese when compared to those breastfed for less than 17 weeks, as were those who had received solid food at 26 weeks or later compared to those who received solid food before 17 weeks, although the latter association was non-significant (Table 2).

After adjusting for covariates and potential confounders, children’s weight status was independently associated with duration of breastfeeding, maternal pre-pregnancy BMI, smoking in pregnancy, and infant birth weight, but not with the age at which solid food was introduced (Table 3). Children whose mothers were obese had more than twice the odds of being overweight/obese (Adjusted Odds Ratio (AOR) 2.12; 95%CI 1.24, 3.62) at 24 to 36 months compared to children of healthy weight mothers. Children whose mothers did not smoke in pregnancy had less than half the odds of being overweight/obese (AOR 0.42; 95%CI 0.19, 0.91) when compared to those whose mothers smoked. Each additional 100g of birth weight was associated with a 6% increase in risk of being overweight/obese (AOR 1.06; 95%CI 1.02, 1.10). Those children who were breastfed for at least the first year of their life had roughly half the odds (AOR 0.48; 95%CI 0.27, 0.87) of being overweight/obese when compared to those children who were never breastfed or had been breastfed for less than 17 weeks. There was a strong inverse relationship (*p* for trend =0.009) and each additional week of breastfeeding was associated with a one percent reduction in risk of being overweight/obese (AOR 0.989; 95%CI 0.981, 0.997). 

There was no significant association with the timing of solid food, and no interaction was found between the early introduction of solid food and the duration of breastfeeding and subsequent weight status. When data were stratified according to the breastfeeding status of children at 17 weeks there was no association between age of introduction of solid food and weight status in those who were breastfed for less than 17 weeks (*p* = 0.561) or those breastfed for 17 weeks or more (*p* = 0.260).

## 4. Discussion

The present study found that children aged 24 to 36 months who were breastfed for at least the first year of their life, as recommended by the current Australian Infant Feeding Guidelines [22], were half as likely to be overweight/obese compared to those who were never breastfed or were breastfed for less than 17 weeks. This relationship was independent of maternal age, socioeconomic status, pre-pregnancy BMI, smoking in pregnancy, method of delivery, infant birth weight and age at which solid food was introduced. There was a strong inverse association, with each additional week of breastfeeding being associated with a 1% decrease in risk of being overweight/obese. This dose response relationship is consistent with the results of other studies [47,48,49], including a meta-analysis that estimated that each additional month of breastfeeding was associated with a 4% decrease in risk of being overweight [50]. 

The relationship between breastfeeding and overweight or obesity in later life has been the subject of numerous systematic reviews of observational studies [7,8,9,10,50], with all concluding that breastfeeding is significantly associated with a reduced risk in later life. A recent systemic review by Horta and colleagues found, that among 11 large, high-quality studies that controlled for socioeconomic status and parental anthropometry, breastfeeding was associated with a 13% reduction in the risk of being overweight/obese [7]. 

A number of potential mechanisms have been postulated for the observed association between breastfeeding and overweight and obesity in childhood and later life [51,52]. There are notable differences in the nutritional composition of breastmilk and infant formula. In particular, infant formula generally has a higher protein content than breastmilk, and protein intakes in excess of metabolic needs in early life may stimulate the secretion of insulin and insulin growth factor type one (IGF-1), which in turn, promotes weight gain in infancy [53]. In a RCT of formula fed infants, increased levels of insulin production and IGF-1 were observed among infants randomised to receive a high protein formula, and total IGF-1 was significantly associated with growth in the first six months of life [54]. Similarly, the infants in a large multicentre RCT randomised to receive a high protein formula had a significantly higher weight for length and BMI at 12 and 24 months of age when compared to those who received a low protein formula or to breastfed infants [55]. 

Breastmilk and infant formula also differ with respect to consistency in composition; the composition of infant formula being constant, whereas the composition of breastmilk varies according to the stage of lactation and between mothers [56]. For example, the concentration of ghrelin, leptin, adiponectin, and insulin in breastmilk changes throughout the postpartum period, and the concentration of some of these hormones are influenced by maternal BMI, particularly for leptin [56,57,58]. These hormones are not present in infant formula but may influence metabolism in early infancy, and program appetite by providing hunger and satiety cues [52,56,59]. In recent times, increasing attention has been paid to the composition of gut microbiota, which is shaped by early feeding experiences [52], and has been associated with obesity [60,61]. There are notable and important differences in the gut microbiota of breastfed and formula fed infants [52], which persist even after the introduction of solid food [62]. It has been proposed that these differences may ‘influence the absorption and storage of energy, linking the nutritive content and bioactive composition of early feeding to infant weight gain and long-term vulnerability to obesity’ [52] (p. 356).

It has also been suggested that behavioural factors could contribute, at least in part, to the observed differences between breastfed and formula fed infants. It has been reported that infants who received breastmilk or infant formula in a bottle gained more weight at one year of age as compared to infants who received breastmilk directly from the breast, thus highlighting that the mode of delivery could be more important than whether the infant is consuming breastmilk or infant formula [63]. This could be due to mothers who breastfeed being less strict with a feeding regime and being more likely to follow the infant’s hunger cues, and infants fed directly from the breast having better appetite regulation than formula fed infants, all of which could influence their weight status later in life [64,65,66]. 

Although the majority of infants receive most breast milk feeds directly from the breast, women in high-income countries are increasingly using breast pumps and bottles to provide their infants with breast milk. Indeed, some ‘breastfeeding’ mothers never feed their infants directly from the breast [67]. In this study, no distinction was made between the modes of delivery by which the breast milk was received. However, given that infants fed breast milk only by a bottle have been shown to gain significantly more weight in the first year of life than infants fed directly from the breast [63], it is important that researchers make this distinction in the future [68].

This present study is one of the first Australian studies to investigate whether children’s weight status at 24 to 36 months was associated with the early introduction of solid food, independent of the duration of breastfeeding. A number of possible mechanisms have been postulated by those studies which have found an association between the early introduction of solid food and risk of overweight/obesity. For instance, the early introduction of solid food, resulting in the cessation of exclusive breastfeeding, may impact appetite control and disturb an infant’s ability to self-regulate their energy intake [23,35,69]. Other studies have reported that the early introduction of solid food was a risk factor for the increased consumption of high energy, sugary, and high fat foods [70,71] in the first year of life. Higher energy intake during the weaning period has been associated with higher BMI in childhood [21].

Only one other Australian study has examined the association of age of introduction of solid food and weight status while controlling for duration of breastfeeding [35]. Unlike this earlier study, which reported that delaying the introduction of solid food reduced the odds of being overweight/obese at 10 years of age by roughly 10% per additional week [35], no independent association between the age of introduction of solid food and weight status was found in this study. However, given the differences in the ages of children when weight status was measured, the findings of the two studies are not directly comparable. Consistent with other researchers [35,72], there was no interaction between breastfeeding duration and age of introduction of solid food which has been reported in other studies [22,23,24]. These findings are consistent with previous systemic reviews that concluded that the age of introduction of solid food was not clearly associated with childhood overweight/obesity [19,20,21]. 

While plausible underlying physiological and behavioural mechanisms for the association between breastfeeding duration and overweight/obesity that is reported in this study have been postulated and are being investigated, the issue of unmeasured and residual confounding remains a possibility [73]. This risk was reduced in this study by controlling for a variety of confounding variables and identified risk factors for childhood obesity not always adjusted for in other studies. These included a number of measures of socio-economic status, infant birth weight, maternal pre-pregnancy BMI, smoking in pregnancy, and method of delivery, all of which have been shown in other studies to be associated with the risk of overweight/obesity in childhood [14]. Additional strengths of this study are that children’s weight and height were measured using standardised procedures and equipment and not based on maternal report, and information on the infant feeding practices of interest was collected prospectively on a number of relatively closely spaced occasions in the first year of life, thus reducing the potential for recall bias that is associated with the retrospective recall of breastfeeding. 

Limitations to this study include that it did not control for behavioural and environmental factors, such as maternal feeding styles and family food practices, which have been demonstrated to be associated with an infant’s weight status [73]. More than half of the children that were recruited into SMILE did not attend the dental examination which meant that the BMI of these children could not be determined, thus reducing the sample size and the generalizability of the findings. While participants that were included in this analysis were more likely to be older, better educated, and more socially advantaged than non-participants, intentional oversampling of mother-infant dyads from socially disadvantaged areas [36] means that the analysis population consisted of a relatively socio-economically diverse cohort of children. 

## 5. Conclusions

This study provides further evidence of an inverse relationship between breastfeeding and risk of overweight/obesity in early childhood and the value of sustained breastfeeding to 12 months and beyond, in terms of healthy weight status in early childhood. This association was independent of socio-economic determinants, maternal pre-pregnancy BMI, smoking in pregnancy, delivery method, infant birth weight, and age at which solid food was introduced. This study contributes to the growing but inconsistent body of evidence investigating the early introduction of solid food as a risk factor for overweight/obesity. No association was found between the age of introduction of solid food and risk of overweight/obesity, nor was there any evidence of an interaction between age of introduction of solid food and the duration of breastfeeding reported in other studies. The promotion of sustained breastfeeding remains an important public health strategy to combat the growing epidemic of childhood obesity.

## Figures and Tables

**Table 1:** Maternal and child characteristics according to different categories of breastfeeding duration.

			Duration of Breastfeeding (Weeks)			
Characteristics	Total		<17 (N = 263)	17–25 (N = 92)	26–51 (N = 221)	≥52 (N = 377)
	(*n*)	%	%	%	%	%
***Maternal characteristics***						
Maternal age at birth (years)						
Mean (SD)	29.9	(5.4)	29.1 (5.7)	29.1 (4.9)	30.4 (4.4)	31.45 (4.7)
<25	(96)	10.1	21.5	9.8	5.9	4.8
25-34	(652)	68.6	60.5	71.7	75.6	69.2
≥35	(203)	21.3	18.0	18.5	18.6	26.0
Maternal level of education						
School/vocational	(414)	43.6	65.3	44.6	40.3	30.2
Some university ^a^ and above	(535)	56.4	34.7	55.4	59.7	69.8
Maternal country of birth						
Australia and New Zealand	(684)	72.3	81.1	68.5	71.0	67.9
United Kingdom/Ireland	(33)	3.5	3.9	6.5	2.3	3.2
India	(67)	7.1	4.6	8.7	8.6	7.5
China	(35)	3.7	1.9	2.2	3.2	5.6
Asia-other	(68)	7.2	3.5	10.9	9.0	7.8
Other	(59)	6.2	5.0	3.3	5.9	8.0
IRSAD ^b^						
Deciles 1–2	(149)	15.7	18.9	18.9	14.9	12.5
Deciles 3–4	(204)	21.5	24.4	24.4	19.0	19.5
Deciles 5–6	(198)	20.9	16.7	16.7	21.7	22.1
Deciles 7–8	(180)	19.0	15.6	15.6	24.0	18.1
Deciles 9–10	(217)	22.9	24.4	24.4	20.4	27.7
Number of children						
1	(442)	47.6	52.1	48.3	49.3	43.2
2	(334)	36.0	29.6	36.0	39.1	38.6
≥3	(153)	16.5	18.3	15.7	11.6	18.2
Maternal pre-pregnancy BMI (kg/m^2^)						
<18.5	(36)	4.0	4.5	1.1	2.4	5.3
18.5–24.99	(479)	53.4	37.7	53.4	53.9	63.8
25–29.99	(208)	23.2	29.1	23.9	27.7	16.4
≥30	(174)	19.4	28.7	26.1	16.0	14.5
Delivery method						
Vaginal	(524)	55.2	49.4	52.2	55.9	59.4
Caesarean section	(426)	44.8	50.6	47.8	44.1	40.6
Smoked in Pregnancy						
Yes	(55)	5.8	11.9	7.6	3.2	2.7
No	(893)	94.2	88.1	92.4	96.8	97.3
***Child characteristics***						
Gender						
Male	(512)	53.8	51.9	63.0	53.4	53.1
Female	(596)	51.7	48.1	37.0	46.6	46.9
Birthweight (grams)						
<2500	(59)	6.3	90.0	94.4	92.3	94.1
2500–4499	(873)	92.6	8.8	4.4	6.4	4.8
≥4500	(11)	1.2	1.2	1.1	1.4	1.1
Age at time of dental exam (months)						
24–29	(533)	55.9	54.0	58.7	51.6	59.2
≥30	(420)	44.1	46.0	41.3	48.4	40.8
Child weight						
Healthy	(843)	88.5	84.0	87.0	86.9	92.8
Overweight	(86)	9.0	12.5	9.8	10.4	5.6
Obese	(24)	2.5	3.4	3.3	2.7	1.6
Age of introduction of solid food (weeks)						
<17	(249)	26.1	45.6	28.3	20.8	15.1
17–25	(616)	64.6	47.5	63.0	72.9	72.1
≥26	(88)	9.2	6.8	8.7	6.3	12.7

^a^ Commenced but did not complete University. ^b^ IRSAD: Index of Relative Socio-Economic Advantage and Disadvantage with decile 1 = most disadvantaged and 10 = most advantaged; BMI: Body Mass Index.

**Table 2:** Unadjusted odds ratios (95%CI) of being overweight/obese (body mass index (BMI) z-score ≥ 2 SD) in children aged 24 to 36 months according to infant feeding practices and maternal and child characteristics.

Explanatory variables	OR	95%CI	*p*-Value
*Maternal characteristics*			
Maternal age at birth (years)	0.96	0.93, 1.00	0.070
Maternal level of education			
School/vocational	1.00		
Some university ^a^ and above	0.75	0.50, 1.11	0.153
Maternal country of birth			
Australia and New Zealand	1.00		
United Kingdom/Ireland	1.33	0.50, 3.54	0.569
India	1.01	0.47, 2.19	0.981
China	0.70	0.21, 2.33	0.559
Asia-other	0.72	0.30, 1.72	0.460
Other	0.84	0.35, 2.02	0.702
IRSAD ^b^			
Deciles 1–2	2.10	1.11, 3.97	0.022
Deciles 3–4	1.01	0.51, 1.98	0.980
Deciles 5–6	1.79	0.97, 3.30	0.063
Deciles 7–8	1.16	0.59, 2.28	0.671
Deciles 9–10	1.00		
Number of children			
1	1.00		
2	0.97	0.61, 1.53	0.892
≥3	1.35	0.79, 2.31	0.280
Maternal pre-pregnancy BMI (kg/m^2^)			
<25	1.00		
25–29.99	1.75	1,05, 2.91	0.031
≥30	2.44	1.48, 4.03	<0.001
Delivery method			
Vaginal	1.00		
Caesarean section	1.10	0.74, 1.64	0.642
Smoked in pregnancy			
Yes	1.00		
No	0.35	0.18, 0.66	0.001
***Child characteristics***			
Gender			
Male	1.00		
Female	0.89	0.60, 1.33	0.564
Birthweight per 100 grams	1.06	1.02, 1.10	0.002
Breastfeeding duration (weeks)			
<17	1.00		
17–25	0.79	0.40, 1.58	0.502
26–51	0.80	0.48, 1.33	0.378
≥52	0.41	0.24, 0.68	0.001
Age of introduction of solid food (weeks)			
<17	1.00		
17–25	0.77	0.50, 1.19	0.243
≥26	0.45	0.18, 1.10	0.081

^a^ Commenced but did not complete University. ^b^ IRSAD: Index of Relative Socio-Economic Advantage and Disadvantage with decile 1 = most disadvantaged and 10 = most advantaged OR: Odds ratio; CI: Confidence interval; BMI: Body mass index.

**Table 3:** Adjusted odds ratio (95%CI) of being overweight/obese (BMI z-score ≥ 2 SD) in children aged 24 to 36 months according to infant feeding practices, maternal BMI, smoking in pregnancy and infant birth weight (*n* = 878).

Explanatory variables	AOR ^a^	95%CI	*p*-Value
Mother BMI			0.003 ^b^
<25	1.0 (reference)		
25–29.99	1.42	0.83, 2.44	0.199
≥30	2.12	1.24, 3.62	0.006
Smoked in pregnancy			
Yes	1.00		
No	0.42	0.19, 0.91	0.027
Birthweight per 100 grams	1.06	1.02, 1.10	0.004
Breastfeeding duration (weeks)			0.009^b^
<17	1.00		
17–25	0.80	0.37, 1.70	0.555
26–51	0.82	0.46, 1.47	0.507
≥52	0.49	0.27, 0.90	0.020
Age of introduction of solid food (weeks)			
<17	1.00		
17–25	0.94	0.57, 1.54	0.799
≥26	0.56	0.20, 1.55	0.262

^a^ Adjusted for maternal age, education and social disadvantage. ^b^ *p* for trend. AOR: Adjusted odds ratio; CI: Confidence interval; BMI: Body mass index

## Supplementary Materials

The following are available online at http://www.mdpi.com/1660-4601/15/4/599/s1, Figure S1: Participant Flow Chart; Table S1: Characteristics of participant and non-participant mothers and infants.
