# Supplementary material for: Duration of Breastfeeding, but Not Timing of Solid Food, Reduces the Risk of Overweight and Obesity in Children Aged 24 to 36 Months: Findings from an Australian Cohort Study
Source: Int J Environ Res Public Health. 2018 Mar 26;15(4):599. doi: 10.3390/ijerph15040599 (PMC5923641; doi:10.3390/ijerph15040599)
Supplement: Supplementary file 1 [file ijerph-15-00599-s001.pdf]

International Journal of
Environmental Research and Public Health

MDPI

Article

Duration of Breastfeeding, but Not Timing of Solid Food, Reduces the Risk of Overweight and Obesity in Children Aged 24 to 36 Months: Findings from an Australian Cohort Study

Sarah Bell [1], Sarah Siau Yi Yew [1], Gemma Devenish [1], Diep Ha [2], Loc Do [2] and Jane Scott [1,*]

Supplementary Materials

Table S1. Characteristics of participant and non-participant mothers and infants.

Characteristics	Participants (n = 953)		Non-participants (n = 1159)		
	n	%	n	%	*p*-value
**Maternal characteristics**					
Maternal age at birth (years)					<0.001 [a]
<25	96	10.1	240	20.9	
25-34	652	68.6	701	61.0	
≥35	203	21.3	209	18.2	
Maternal level of education					<0.001 [b]
School/vocational	414	43.6	722	62.5	
Some university and above	535	56.4	433	37.5	
Maternal country of birth					
Australia and New Zealand	684	72.3	769	66.8	0.016 [a]
United Kingdom/Ireland	33	3.5	44	3.8	
India	67	7.1	119	10.3	
China	35	3.7	30	2.6	
Asia-other	68	7.2	107	9.3	
Other	59	6.2	82	7.1	
IRSAD [c]					<0.001 [a]
Deciles 1-2	149	15.7	313	27.7	
Deciles 3-4	204	21.5	239	21.2	
Deciles 5-6	198	20.9	192	17.0	
Deciles 7-8	180	19.0	204	18.1	
Deciles 9-10	217	22.9	181	16.0	
Number of children					0.229 [a]
1	442	47.6	419	44.9	
2	334	36.0	334	35.8	
≥3	153	16.5	181	19.4	
Maternal pre-pregnancy BMI (kg/m^2)					0.117 [a]
<18.5	36	4.0	61	5.9	
18.5 – 24.99	479	53.4	508	49.1	
25 – 29.99	208	23.2	247	23.9	
≥30	174	19.4	218	21.1	
Infant characteristics					
Gender					0.357 [b]
Male	512	53.8	596	51.7	
Female	596	51.7	556	48.3	
Birthweight (grams)					0.390 [a]

International Journal of Environmental Research and Public Health

< 2500	59	6.3	83	7.3
2500-4499	873	92.6	1034	91.0
≥ 4500	11	1.2	19	1.7

[a] Chi Square *p* value; [b] Fishers Exact test; [c] IRSAD: Index of Relative Socio-Economic Advantage and Disadvantage with decile 1= most disadvantaged and 10= most advantaged; BMI: Body Mass Index

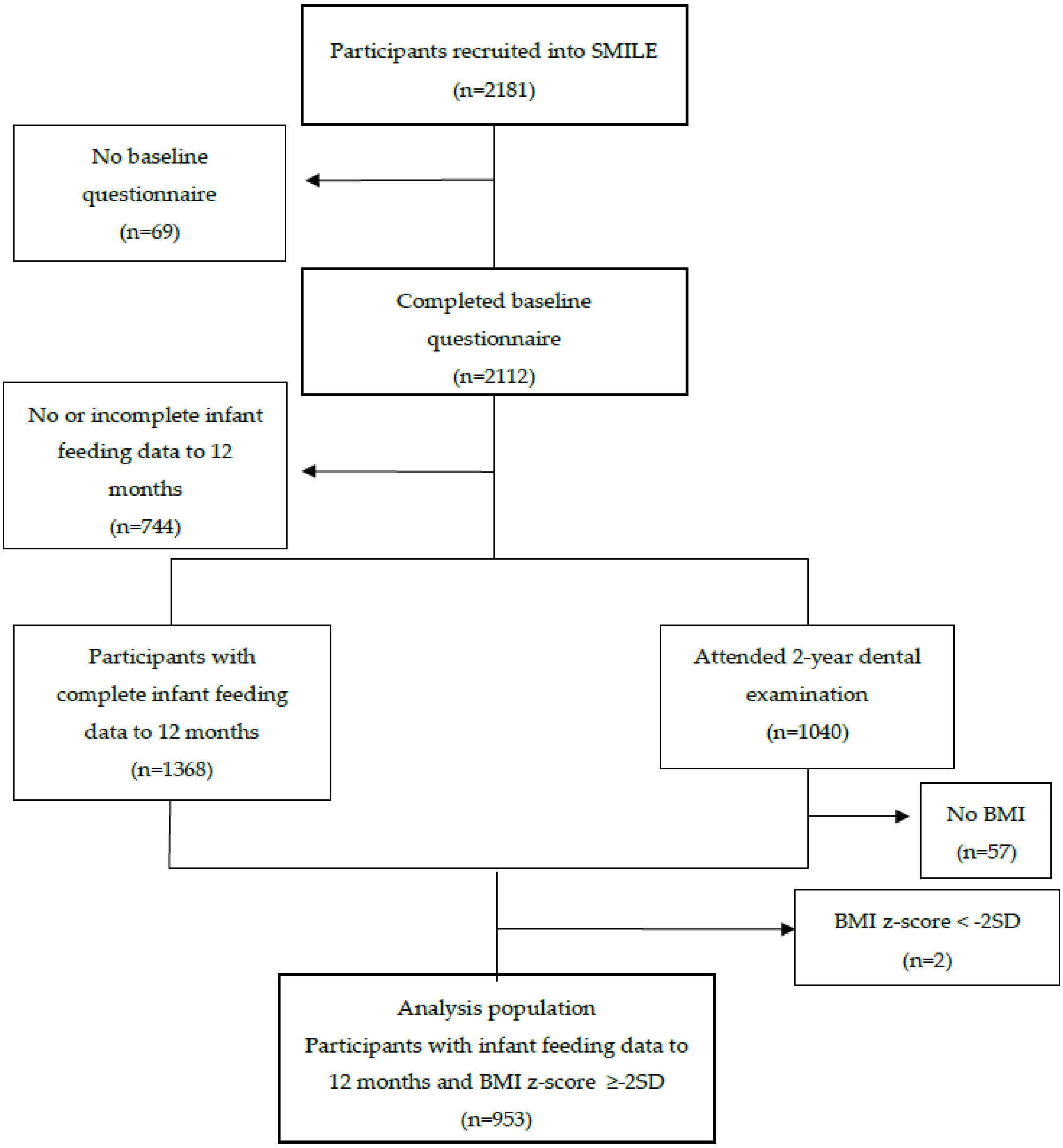

Figure S1. Participant Flow Chart.
